# Long-acting parathyroid hormone receptor agonist rectifies hypocalcemia in autosomal dominant hypocalcemia type 1 mice

**DOI:** 10.1172/JCI201759

**Published:** 2026-02-19

**Authors:** Fadil M. Hannan, Mark Stevenson, Taha Elajnaf, Hussam Rostom, Kate E. Lines, Michelle Stewart, Sara Wells, Lee Moir, Thomas J. Gardella, Rajesh V. Thakker

**Affiliations:** 1 Nuffield Department of Women’s and Reproductive Health, and; 2 Radcliffe Department of Medicine, University of Oxford, Oxford, United Kingdom.; 3 Mary Lyon Centre, MRC Harwell, Harwell Science and Innovation Campus, Oxfordshire, United Kingdom.; 4 Endocrine Unit, Massachusetts General Hospital, and Harvard Medical School, Boston, Massachusetts, USA.; 5 William Harvey Research Institute, London School of Medicine, Queen Mary University of London, London, United Kingdom.

**Keywords:** Bone biology, Endocrinology, Calcium

## Abstract

Eneboparatide, a long-acting modified form of parathyroid hormone, increases serum calcium levels without increasing urine calcium in mice with autosomal dominant hypocalcemia type 1

**To the Editor:** Autosomal dominant hypocalcemia type 1 (ADH1) is due to gain-of-function calcium-sensing receptor (CaSR) mutations that cause hypocalcemia, hyperphosphatemia, hypomagnesemia, low parathyroid hormone (PTH), and hypercalciuria (1). Calcium and vitamin D analogs represent first-line therapy for symptomatic ADH1 but predispose individuals to nephrocalcinosis and renal impairment (1). CaSR antagonists constitute a targeted ADH1 therapy but are not yet clinically approved (2). Recombinant PTH is used to manage ADH1 symptoms but is limited, as multiple daily injections or continuous infusion are required (1). We investigated eneboparatide (EPT), a long-acting PTH receptor 1 (PTH1R) agonist, as an ADH1 therapy. EPT binds to a PTHR1 conformation, that prolongs cyclic AMP signaling and induces sustained calcemic actions (3). EPT increases serum calcium in rats and patients with hypoparathyroidism without causing hypercalciuria (3, 4). However, its potential to treat ADH1 is unclear. We assessed this using nuclear flecks (*Nuf*) mice, an ADH1 model harboring a gain-of-function CaSR mutation, Leu723Gln (2). Heterozygous (*Casr^+/Nuf^*) and homozygous (*Casr^Nuf/Nuf^*) mice were used as moderate and severe ADH1 models, respectively (2).

We assessed the duration of EPT action in WT and *Casr^+/Nuf^* mice. EPT was administered as a once-daily 2 nmol/kg s.c dose, based on hypoparathyroid rat studies (3). Mice were treated for 14 days to achieve steady-state calcemic responses, and ionized calcium was assessed before the dose and 6–24 hours after the dose on day 14. WT and *Casr^+/Nuf^* mice showed peak ionized calcium increases at 6 hours, with values returning to baseline by 24 hours (Figure 1, A–D). Next, we performed 14-day dose-ranging in *Casr^+/Nuf^* and *Casr^Nuf/Nuf^* mice and assessed the effects of once-daily vehicle, 1, 2, or 4 nmol/kg EPT on plasma mineral levels 6 hours after the final dose. At baseline, *Casr^+/Nuf^* and *Casr^Nuf/Nuf^* mice were hypocalcemic, with adjusted calcium levels of 1.77 ± 0.04 and 1.65 ± 0.07 mmol/L, respectively (physiological range = 2.33–2.63 mmol/L) (Figure 1E and Supplemental Figure 1; supplemental material available online with this article; https://doi.org/10.1172/JCI201759DS1) (2). EPT exerted dose-dependent effects, with the 2 nmol/kg dose resulting in near-normal adjusted calcium levels of 2.24 ± 0.04 and 2.33 ± 0.5 mmol/L in *Casr^+/Nuf^* and *Casr^Nuf/Nuf^* mice, while 4 nmol/kg caused hypercalcemia (Figure 1E and Supplemental Figure 1). EPT did not alter phosphate, magnesium, or the calcium x phosphate product (Figure 1, F–H, and Supplemental Figure 1). However, EPT increased 1,25-dihydroxyvitamin D [1,25(OH)_2_D] and alkaline phosphatase (ALP) and caused dose-dependent increases of the procollagen type 1 N-terminal propeptide (P1NP) and C-terminal telopeptide of type 1 collagen (CTX-1) bone turnover markers, which correlated with the adjusted calcium levels (Figure 1, I–P, and Supplemental Figure 1).

We analyzed fractional excretion (FE) in mice receiving vehicle or 2 nmol/kg EPT. Fourteen days of treatment did not alter calcium FE, but it decreased magnesium FE in *Casr^+/Nuf^* and *Casr^Nuf/Nuf^* mice and increased phosphate FE in *Casr^+/Nuf^* mice (Figure 1, Q–S, Supplemental Figure 1, and Supplemental Table 1). These effects were not associated with altered kidney expression of the *Casr* and *Pth1r* genes or of genes mediating calcium and magnesium reabsorption (*Slc12a1*, *Kcnj1*, *Trpv5*, *Cldn16*, *Cldn19*) or phosphate excretion (*Slc34a1*, *Slc34a3*) (Supplemental Figure 2). Dual-energy x-ray absorptiometry in *Casr^+/Nuf^* mice receiving vehicle or 2 nmol/kg EPT for 14 days revealed bone mineral density (BMD) decreases in the treated mice (Figure 1T and Supplemental Table 2).

In summary, EPT rectified the hypocalcemia in *Nuf* mice in a dose-dependent manner, consistent with its effects in rats and patients with hypoparathyroidism (3, 4). EPT increased adjusted calcium levels without causing hypercalciuria, probably by promoting renal calcium reabsorption (Supplemental Figure 3). This contrasts with vitamin D analogs, which, by elevating circulating calcium, suppress PTH and exacerbate renal CaSR activation, thereby inhibiting calcium reabsorption (Supplemental Figure 3) (1, 5). EPT potentially altered mineral excretion posttranscriptionally by inducing the translocation of proximal tubule sodium-phosphate cotransporters or claudins in the cortical thick ascending limb. The lack of effect of EPT on plasma phosphate and urinary calcium levels in *Casr^+/Nuf^* mice contrasted with these parameters being reduced in patients with hypoparathyroidism treated with EPT and was possibly due to the limited treatment duration and number of mice studied (4).

The calcemic effects of EPT were likely mediated by higher bone turnover and increased 1,25(OH)_2_D, as both correlated with adjusted calcium levels. EPT induced similar increases in P1NP and CTX-1 expression, consistent with the augmentation of balanced bone turnover reported in patients with hypoparathyroid (4). However, the increased bone turnover in patients and rats with hypoparathyroidism treated with EPT was mild and did not alter BMD (3, 4), whereas *Nuf* mice showed pronounced increases in bone turnover and decreased BMD. These skeletal effects of EPT were potentially due to disease- or species-specific differences and warrant evaluation in patients with ADH1. This study demonstrates the potential of EPT for managing ADH1.

## Funding support

- Wellcome Trust Investigator Award (106995/Z/15/Z).

## Supplementary Material

Supplemental data

Supporting data values

## Figures and Tables

**Figure 1:**
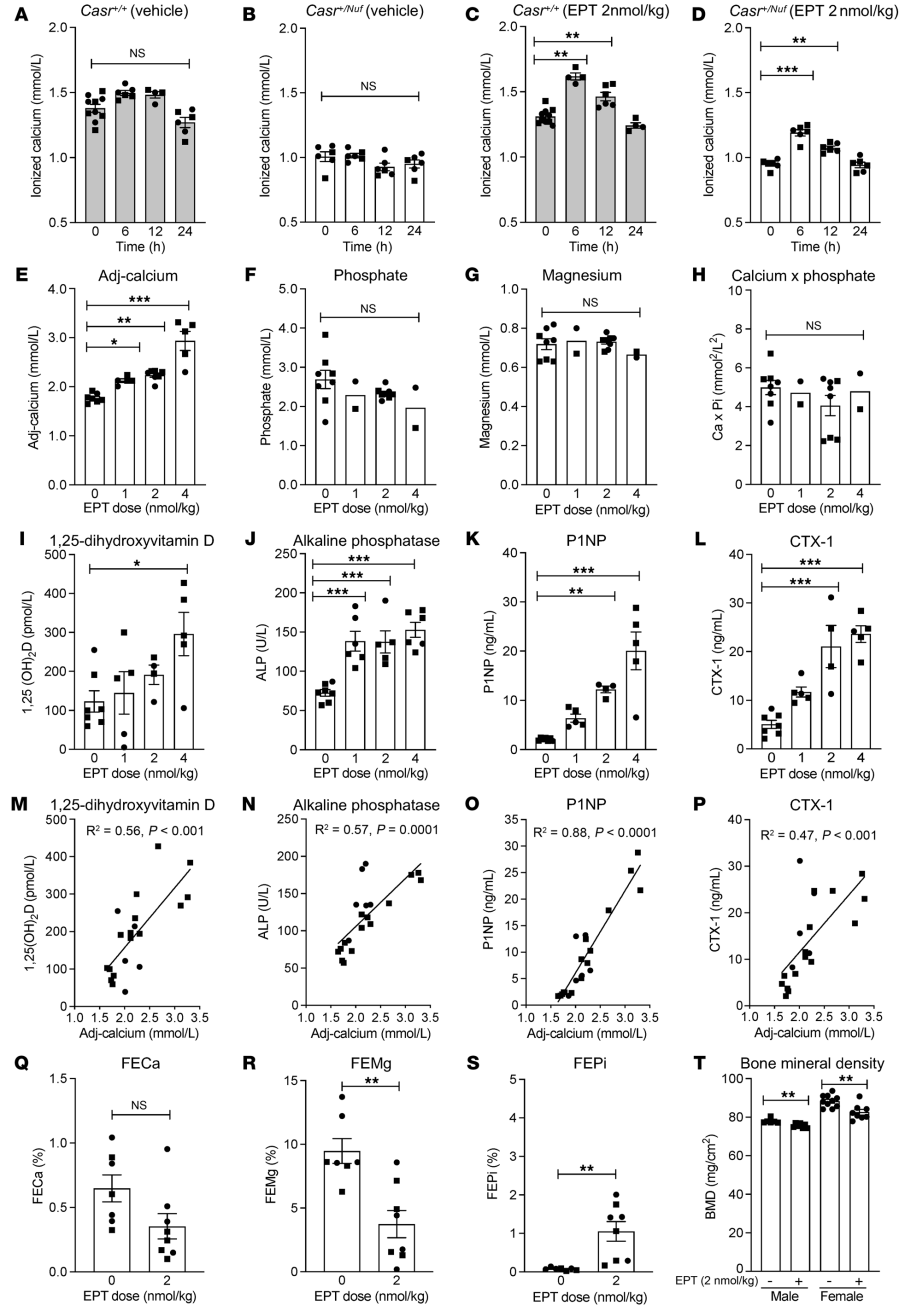
Mineral and bone effects of EPT. (**A**–**D**) Blood ionized calcium alterations in (**A**) *Casr^+/+^* and (**B**) *Casr^+/Nuf^* mice treated with vehicle and in (**C**) *Casr^+/+^* and (**D**) *Casr^+/Nuf^* mice treated with 2 nmol/kg EPT. (**E**–**L**) Effect of 1–4 nmol/kg EPT on plasma concentrations of (**E**) adjusted calcium (Adj-calcium), (**F**) phosphate, (**G**) magnesium, (**H**) calcium x phosphate, (**I**) 1,25(OH)_2_D, (**J**) ALP, (**K**) P1NP, and (**L**) CTX-1. (**M**–**P**) Association of adjusted calcium with (**M**) 1,25(OH)_2_D, (**N**) ALP, (**O**) P1NP, and (**P**) CTX-1. (**Q**–**S**) FE of (**Q**) calcium (FECa), (**R**) magnesium (FEMg), and (**S**) phosphate (FEPi). (**T**) BMD of male and female *Casr^+/Nuf^* mice treated with vehicle (–) or 2 nmol/kg EPT (+). Mean ± SEM values are shown in the bar charts and scatter plots. Shaded and open bars represent *Casr^+/+^* and *Casr^+/Nuf^* mice, respectively. Squares denote male mice; circles denote female mice. *P < 0.05, **P < 0.01, and ***P < 0.001. Two-group comparisons were assessed by 2-tailed Student’s *t* test and comparison of 3 or more groups by 1-way ANOVA.

## Version 1 02/19/2026

In-Press Preview

## Version 2 04/01/2026

Electronic publication
