# Supplementary material for: Long-acting parathyroid hormone receptor agonist rectifies hypocalcemia in autosomal dominant hypocalcemia type 1 mice
Source: J Clin Invest. 2026 Feb 19;136(7):e201759. doi: 10.1172/JCI201759 (PMC13038194; doi:10.1172/JCI201759)
Supplement: Supplemental data [file jci-136-201759-s112.pdf]

Supplemental Material

Sex as a biological variable

Male and female mice were used in the experiments described in this study. Data from male and female mice were combined for all analyses with the exception of bone mineral parameters, which are shown as separate male and female data due to sex-specific differences.

Author contributions

RVT and TJG conceived the study. FMH and RVT designed the methodology. MS, TE, HR, KEL, MS, SW and LM conducted the investigations. TE, HR, MS, LM and FMH curated the data. FMH performed the formal data analysis. FMH and RVT wrote the original manuscript draft. RVT had responsibility for the research activity, planning and execution. All authors critically reviewed and revised the draft manuscript, and also approved the final version for publication.

Acknowledgements

We gratefully acknowledge Amolyt Pharma for providing eneboparatide. This work was supported by funding from a Wellcome Trust Investigator Award (grant number 106995/Z/15/Z) (to RVT).

Methods

Compounds. Eneboparatide, also referred to as LA-PTH or AZP-3601, was supplied by Amolyt Pharma and Dr Thomas Gardella. This peptide was diluted in a citrate buffered saline vehicle, as reported (1). The vehicle was prepared by adding 1,753.2 mg of NaCl and 192.1 mg of citric acid to 75 mL of double distilled H_2O (ddH_2O). This was adjusted to pH 5.0 using 10N NaOH, and ddH_2O added to achieve a total volume of 100 mL of citrate buffered saline. Tween 80 (100 µL) was then added and the vehicle filtered through a 0.25 µM Stericup vacuum filtration system prior to use.

Animals. Nuf mice (symbol: $Casr^{Nuf}$; Mouse Genome Informatics (MGI) ID: MGI:3054788) were maintained as a closed colony on the inbred 102/H background (MGI:5291924), which is a substrain

originally bred at the Medical Research Council (MRC) Harwell Centre (2). Mice were kept according to UK Home Office guidance at the MRC Harwell Centre in an environment controlled for temperature (21 ± 2°C), humidity (55 ± 10%) and light (12 hours light-dark cycle) (2). Mice were fed *ad libitum* on a commercial diet (RM3, Special Diet Services) that had 1.24% calcium, 0.83% phosphorus and 2948 IU/kg of vitamin D, and were also given free access to water (25 ppm chlorine) (2). Study mice were 16-35 weeks old and age-matched for all comparisons.

Eneboparatide administration. Adult male and female wild-type ($Casr^{+/+}$), heterozygous ($Casr^{+/Nuf}$) and homozygous ($Casr^{Nuf/Nuf}$) mice were randomly allocated to receive bolus subcutaneous (*s.c*) doses of the vehicle or eneboparatide. No experimental procedures were conducted on the mice prior to drug administration. The study team were blinded during drug dosing, sample collection and when performing end-point assays. The primary study outcome was an increase in plasma albumin-adjusted calcium at 6 hours after drug administration in $Casr^{+/Nuf}$ mice.

Whole blood and plasma biochemical analysis. Whole blood was obtained from the lateral tail vein for ionized calcium measurements using RAPIDLyte Multicap-S plastic blood collection capillary tubes (Siemens Healthineers, Germany), following administration of topical local anaesthesia. Blood ionized calcium was immediately analyzed using the RAPIDLab 348EX Blood Gas System (Siemens Healthineers, Germany), as reported (3). Blood was also obtained from the retro-orbital vein under isoflurane terminal anaesthesia using lithium heparin capillary blood collection tubes (Sarstedt) for other biochemical analyses (2, 4). Samples were centrifuged at 5000 g for 10 min at 8°C, and plasma separated for analysis of total calcium, albumin, phosphate, magnesium, alkaline phosphatase activity, sodium, potassium, and creatinine on a Beckman Coulter AU680 analyzer, as reported (2, 4). Plasma calcium was adjusted for variations in plasma albumin as follows: (plasma calcium (mmol/l) – [(plasma albumin (g/l) – 30) x 0.02] (2, 4). The 1,25-dihydroxyvitamin D metabolite was assayed by a two-step process involving purification by immunoextraction and quantification by enzyme immunoassay (Immunodiagnostic Systems) (4). C-terminal cross-linking telopeptide of type 1 collagen (CTX-1) was measured using a mouse-specific ELISA (BIorbyt Ltd) (4). Procollagen type 1

N-terminal propeptide (P1NP) was measured by an enzyme immunoassay (EIA) (Immunodiagnostic Systems) (5).

Urine biochemistry analysis. Mice were individually housed in metabolic cages (Techniplast), and fed *ad libitum* on water and powdered chow. Mice were allowed to acclimatise to their environment over a 72h period, as described, prior to collection of 24h urine samples and plasma samples (6). Urine and plasma was analyzed for calcium, phosphate, magnesium, sodium, potassium and creatinine on a Beckman Coulter AU680 analyzer (6). The fractional excretion of calcium, phosphate, magnesium, sodium and potassium were calculated using the formula $U_x/P_x*P_{Cr}/U_{Cr}$, where U_x is the urinary concentration of the filtered substance (substance x) in mmol/L, P_x is the plasma concentration of substance x in mmol/L, U_{Cr} is the urinary concentration of creatinine in mmol/L, and P_{Cr} is the plasma concentration of creatinine in mmol/L (6).

Skeletal imaging. Bone mineral content and bone mineral density were measured in mice by whole body dual energy X-ray absorptiometry (DEXA) scanning, which was performed on mice anesthetized by inhaled isoflurane and using a Lunar Piximus densitometer (GE Medical Systems), as reported (6). DEXA images were analyzed using Piximus software (6).

Renal gene expression analysis. Kidneys were collected from *Casr*$^{+/Nuf}$ and *Casr*$^{Nuf/Nuf}$ male mice, snap frozen in liquid nitrogen, and then stored at -80°C until analysis. RNA was extracted from the upper half of the left kidney using the Bead Mill 4 homogeniser (Fisherbrand, USA). Briefly, kidneys were placed in 7-ml tubes pre-filled with 1.4mm ceramic beads containing pre-chilled RNA lysis buffer (Qiagen, Germany) and subjected to two 15-second homogenisation rounds. Lysates were then used for total RNA extraction using the RNeasy Plus Kit (Qiagen, Germany). RNA concentration and purity were measured using a NanoDrop 1000 spectrophotometer (LabTech International, UK) and all samples had A260/A280 ratios between 1.8–2.0. RNA integrity was assessed by agarose gel electrophoresis and visualised by E-Gel (Thermo Fisher Scientific, USA). All samples had intact and distinct 28s and 18s ribosomal RNA bands. Total RNA (1 µg) was reverse transcribed into cDNA

using the High-Capacity cDNA Reverse Transcription Kit (ThermoFisher Scientific, USA). Synthesized cDNA was diluted (1:15) in RNase-free water and used for subsequent amplification. Gene expression was assessed by real-time qPCR using validated predesigned mouse primers for the *Casr*, *Pth1r, Slc12a1, Kcnj1, Trpv5, Cldn16, Cldn19, Slc34a1 and Slc34a3* genes (Sigma, USA). All primers were used at a working concentration of 10 µM. qPCR reactions were undertaken in 96-well plates using PowerUP SYBR Green PCR master mix (Applied Biosystems, USA) in a final volume of 10µL and conducted on the QuantStudio 3 real-time PCR system (Applied Biosystems, USA). All samples were analysed in technical triplicates and mean threshold cycle (Ct) values were determined using Design and Analysis software (Thermo Fisher Scientific, USA). Mean Ct values for each sample were normalized to the geometric mean of the housekeeping genes (*Actb, B2m, Ywhaz)* and expressed as fold-change relative to Nuf Het vehicle control using the $2^{\wedge-\Delta\Delta Ct}$ method, as reported (7).

Statistics

Analysis of all experimental data was conducted using GraphPad Prism (v.10.4.2) (GraphPad Software). Two group comparisons were assessed by two-tailed Student's t test and one-way ANOVA with Dunnett's multiple comparisons test used for comparison of three or more groups. Associations between continuous variables was evaluated using simple linear regression. Data for continuous variables are shown as mean ± SEM. $p<0.05$ was considered to be statistically significant for all analyses.

Study approval

All procedures involving mice were approved by the MRC Harwell Institute Ethical Review Committee, and licensed under the Animal (Scientific Procedures) Act 1986, which was issued by the UK Home Office (PPL30/2752).

Data availability

The authors declare that all data supporting the findings of this study are available within the paper and its Supplementary Information. The raw data for the main figure and supplemental figures and tables are provided in the accompanying supporting data values file.

References

1. Shimizu M, Joyashiki E, Noda H, Watanabe T, Okazaki M, Nagayasu M, et al. Pharmacodynamic Actions of a Long-Acting PTH Analog (LA-PTH) in Thyroparathyroidectomized (TPTX) Rats and Normal Monkeys. *J Bone Miner Res.* 2016;31(7):1405-12.
2. Hannan FM, Gorvin CM, Babinsky VN, Olesen MK, Stewart M, Wells S, et al. Calcilytic NPSP795 Increases Plasma Calcium and PTH in an Autosomal Dominant Hypocalcemia Type 1 Mouse Model. *JBMR Plus.* 2020;4(10):e10402.
3. Bi R, Fan Y, Lauter K, Hu J, Watanabe T, Cradock J, et al. Diphtheria Toxin- and GFP-Based Mouse Models of Acquired Hypoparathyroidism and Treatment With a Long-Acting Parathyroid Hormone Analog. *J Bone Miner Res.* 2016;31(5):975-84.
4. Hannan FM, Stevenson M, Bayliss AL, Stokes VJ, Stewart M, Kooblall KG, et al. Ap2s1 mutation causes hypercalcaemia in mice and impairs interaction between calcium-sensing receptor and adaptor protein-2. *Hum Mol Genet.* 2021;30(10):880-92.
5. Gorvin CM, Loh NY, Stechman MJ, Falcone S, Hannan FM, Ahmad BN, et al. Mice with a Brd4 Mutation Represent a New Model of Nephrocalcinosis. *J Bone Miner Res.* 2019;34(7):1324-35.
6. Howles SA, Hannan FM, Gorvin CM, Piret SE, Paudyal A, Stewart M, et al. Cinacalcet corrects hypercalcemia in mice with an inactivating Galpha11 mutation. *JCI Insight.* 2017;2(20).
7. Livak KJ, and Schmittgen TD. Analysis of relative gene expression data using real-time quantitative PCR and the $2^{-\Delta\Delta CT}$ method. *methods.* 2001;25(4):402-8.

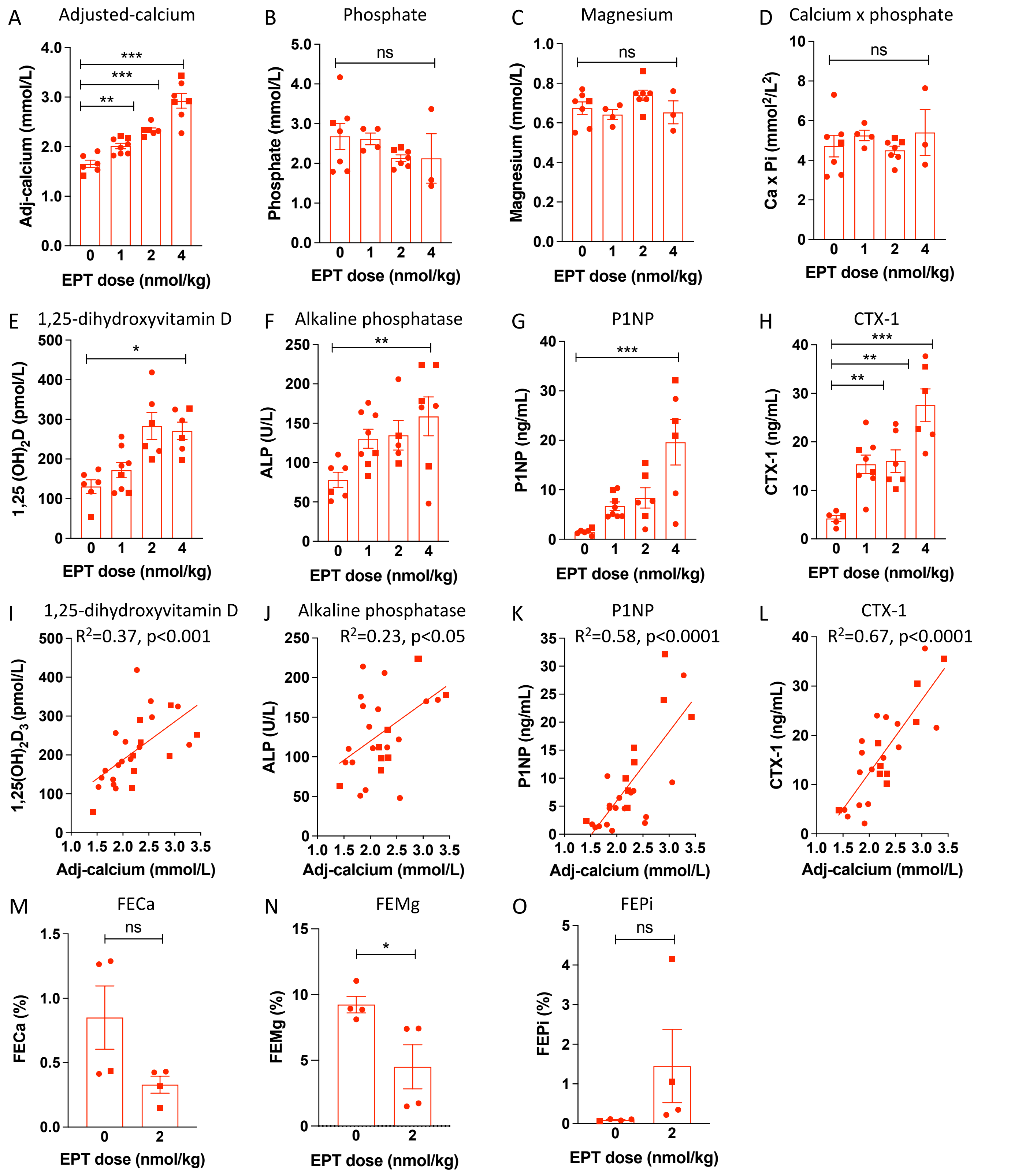

Supplemental Figure 1. Dose-dependent effects of eneboparatide (EPT) in *Casr*$^{Nuf/Nuf}$ mice. (A-D) Dose-dependent effects of 1-4nmol/kg eneboparatide on plasma concentrations of: **(A)** Adjusted-calcium; **(B)** Phosphate; **(C)** Magnesium; **(D)** Calcium x phosphate product; **(E)** 1,25-dihydroxyvitamin D; **(F)** Alkaline phosphatase activity; **(G)** Procollagen type 1 N-terminal propeptide (P1NP); and **(H)** C-terminal telopeptide of type 1 collagen (CTX-1). **(I-L)** Association of adjusted-calcium with: **(I)** 1,25-dihydroxyvitamin D; **(J)** Alkaline phosphatase activity; **(K)** Procollagen type 1 N-terminal propeptide (P1NP); and **(L)** C-terminal telopeptide of type 1 collagen (CTX-1). **(M-O)** Fractional excretion of **(Q)** Calcium (FECa); **(R)** Magnesium (FEMg); and **(S)** Phosphate (FEPi). Mean±SEM values are shown in all hybrid bar chart/scatter plots. Squares, males; circles, females. EPT, eneboparatide. ns, non-significant; *p<0.05; **p<0.01; ***p<0.001. Two group comparisons assessed by 2-tailed Student's *t* test and one-way ANOVA with Dunnett's multiple comparisons test was used for comparison of three or more groups.

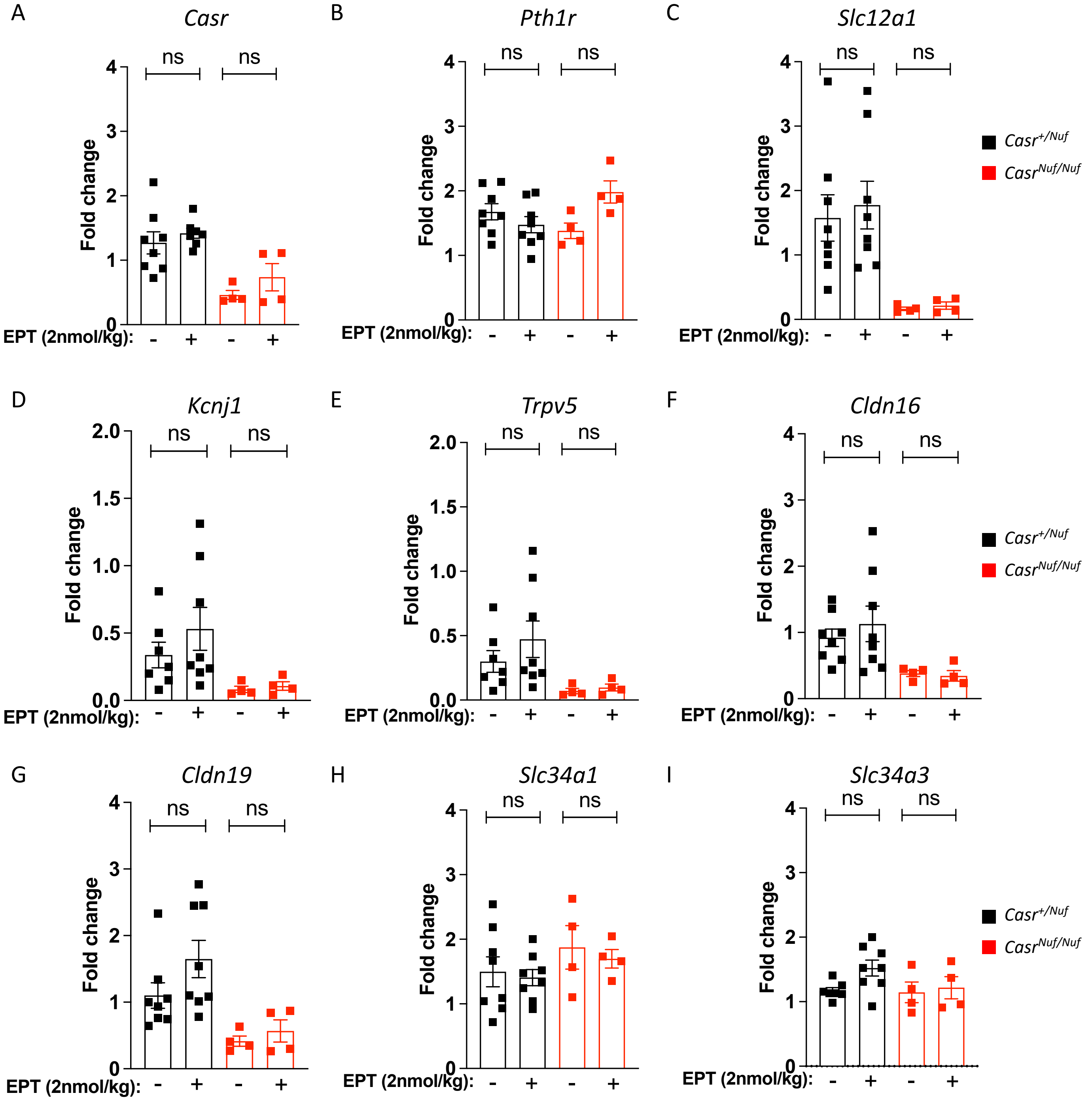

Supplemental Figure 2. Effect of eneboparatide administration on renal expression of genes mediating calcium and magnesium reabsorption or phosphate excretion in male *Casr*$^{+/Nuf}$ and *Casr*$^{Nuf/Nuf}$ mice. Fold-change expression of: (A) *Casr*; (B) *Pth1r*; (C) *Slc12a1*; (D) *Kcnj1*; (E) *Trpv5*; (F) *Cldn16*; (G) *Cldn19*; (H) *Slc34a1;* and (I) *Slc34a3* genes are shown in *Casr*$^{+/Nuf}$ (black) and *Casr*$^{Nuf/Nuf}$ mice (red). +, 2nmol/kg eneboparatide (EPT) administered once-daily for 14 days; -, vehicle-only administered once-daily for 14 days. Mean±SEM values are shown for groups of n=4-8 mice aged 24-43 weeks. ns, non-significant; *p<0.05, **p<0.01, ***p<0.001. Two-tailed Student's *t* test was used for all analyses.

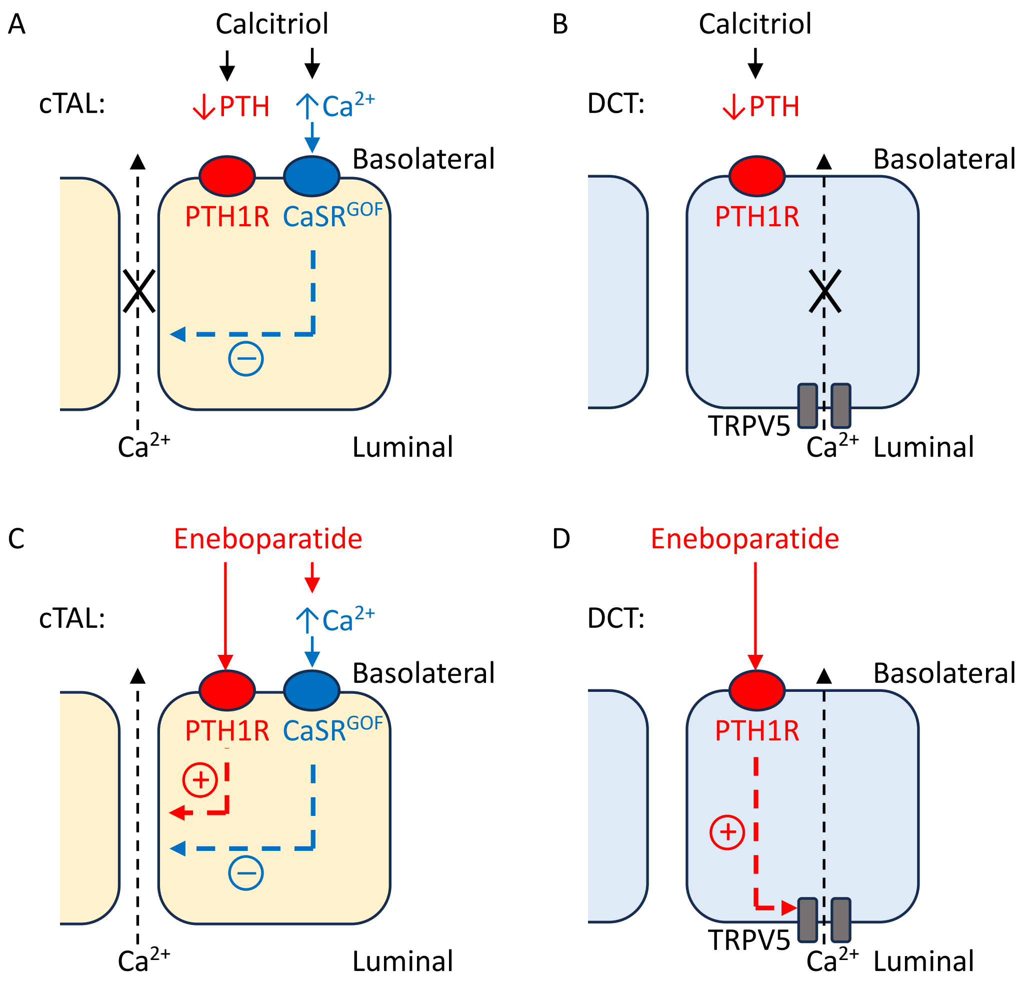

Supplemental Figure 3. Comparison of effects of calcitriol and eneboparatide on urinary calcium reabsorption. (A) In ADH1 patients, calcitriol treatment increases serum calcium (Ca^{2+}), which exacerbates calcium-sensing receptor (CaSR) gain-of-function (GOF) in the cortical thick ascending limb (cTAL) of the Loop of Henle, thereby inhibiting paracellular urinary Ca^{2+} reabsorption. The calcitriol induced rise in serum calcium Ca^{2+} also suppresses parathyroid hormone (PTH) secretion, thus inhibiting PTH-mediated Ca^{2+} reabsorption in the cTAL. (B) Calcitriol induced suppression of PTH secretion also inhibits transcellular Ca^{2+} reabsorption in the distal convoluted tubule (DCT), which is mediated by transient receptor potential cation channel subfamily V member 5 (TRPV5). (C-D) Eneboparatide increases serum Ca^{2}, which similar to calcitriol, may exacerbate CaSR GOF in the cTAL. However, this is counterbalanced by eneboparatide-mediated activation of the PTH-1 receptor (PTH1R) in the cTAL and DCT, which promotes Ca^{2+} reabsorption. ↑, increase; ↓, decrease.

Supplemental Table 1. Effect of eneboparatide on urine volume and biochemistry of *Casr*$^{+/Nuf}$ and *Casr*$^{Nuf/Nuf}$ mice.

	Casr$^{+/Nuf}$		***Casr***$^{Nuf/Nuf}$	
Treatment	Vehicle	EPT 2nmol/kg	Vehicle	EPT 2nmol/kg
N	7 (3M, 4F)	8 (4M, 4F)	4 (1M, 3F)	4 (2M, 2F)
Age (weeks)	34.9±0.2	34.9±0.1	35.0±0.2	35.2±0.1
24hr urine volume (mL)	6.3±0.6	5.5±0.3	6.3±0.7	6.8±1.1
Urine calcium/creatinine ratio[a]	1.0±0.1	0.8±0.2	1.2±0.4	0.5±0.1
24hr urine calcium (µmol/24hr)	16.6±2.8	11.2±2.4	19.1±4.1	5.8±1.0*
FE Calcium (%)	0.65±0.1	0.35±0.1	0.85±0.2	0.33±0.1
FE Phosphate (%)	0.08±0.01	1.1±0.3**	0.09±0.01	1.45±0.9
FE Magnesium (%)	9.5±1.0	3.7±1.1**	9.2±0.6	4.5±1.7*
FE Sodium (%)	0.5±0.02	0.4±0.2	0.4±0.03	0.4±0.04
FE Potassium (%)	25.2±2.4	20.7±0.9	29.0±3.5	23.5±3.4

Urine volume and biochemistry assessed following administration of once-daily subcutaneous bolus of 2 nmol/kg eneboparatide (EPT) or vehicle-only for 14 days. [a]Urine calcium/creatinine ratio shown as mmol/mmol. All values shown as mean±SEM. FE, fractional excretion; M, male; F, female. *p<0.05 and **p<0.01 for a comparison of eneboparatide-treated mice vs. respective vehicle-only treated mice. Two group comparisons assessed by 2-tailed Student's *t* test.

Supplemental Table 2. Effect of eneboparatide on DEXA parameters of *Casr*$^{+/Nuf}$ mice.

	***Casr*$^{+/Nuf}$ males**		***Casr*$^{+/Nuf}$ females**	
Treatment	Vehicle	EPT 2nmol/kg	Vehicle	EPT 2nmol/kg
N	6	7	10	8
Age (weeks)	25.5±0.5	25.7±0.4	25.4±0.3	25.5±0.4
Body weight (g)	32.9±0.8	33.5±0.9	27.3±0.7	26.3±0.9
Fat mass (%)	19.4±0.9	20.4±1.0	16.4±0.7	17.3±1.1
Bone mineral content (mg)	799±13	765±7*	868±20	804±31
Bone mineral content/body weight (mg/g)	24.3±0.4	22.9±0.4*	31.8±0.2	30.5±0.7
Bone mineral density (mg/cm^2)	78±0.5	75.6±0.4**	88.3±1.0	82.6±1.4**

Whole body dual energy X-ray absorptiometry (DEXA) assessed following administration of once-daily subcutaneous bolus of 2 nmol/kg eneboparatide (EPT) or vehicle-only for 14 days. Data shown as mean±SEM. *p<0.05 and **p<0.01 for a comparison of eneboparatide-treated vs. vehicle treated *Casr*$^{+/Nuf}$ mice. Two group comparisons assessed by 2-tailed Student's *t* test.
